# Awareness of Climate Change and Its Impact on Reproductive Health

**DOI:** 10.7759/cureus.116125

**Published:** 2026-09-12

**Authors:** Kalyani Rath, Pritha Chakraborty, Biswakalpita Swain, Rojalin Dash, Anusuya Behera

**Affiliations:** 1 Obstetrics and Gynecology, Kalinga Institute of Medical Sciences (KIMS), Bhubaneswar, IND; 2 Obstetrics and Gynecology, Kalinga Institute of Nursing Sciences (KINS), Bhubaneswar, IND; 3 Obstetrics and Nursing, Kalinga Institute of Nursing Sciences (KINS), Bhubaneswar, IND

**Keywords:** awareness, climate change, knowledge, nursing students, reproductive health

## Abstract

Background and aim

Climate change poses a serious threat and crisis to health globally, with reproductive and maternal health falling under the vulnerable group exposed to extreme temperatures, pollution, and climate-related health crises. Student nurses are the future of healthcare professionals; therefore, it is essential for nursing students to address and cope with these emerging environmental health challenges. This study aimed to assess the awareness of climate change and its impact on reproductive health among nursing students at a selected nursing college in Bhubaneswar, Odisha.

Materials and methods

A descriptive cross-sectional study was conducted with 182 nursing students from various nursing courses, such as B.Sc. Nursing, GNM Nursing, and M.Sc. Nursing, at the Kalinga Institute of Nursing Sciences (KINS), Bhubaneswar. The data were collected using a semi-structured tool with multiple-choice questions that included various sociodemographic data and assessed knowledge and awareness levels related to climate change and its impact on reproductive health. Data were analyzed using SPSS version 25 (Armonk, NY: IBM Corp.) with descriptive statistics and a chi-square test to determine the associations related to it.

Results

Overall, 48.4% of nursing students had good knowledge, 28.0% had average knowledge, and 23.6% had poor knowledge. Statistical analysis revealed that sociodemographic factors such as age, gender, residence, parental occupation, and education were not significantly associated with knowledge levels (all p>0.05). However, courses such as B.Sc. Nursing fifth and sixth semesters and students who had prior exposure to the topic had the highest rate of good knowledge (70%). Students with prior exposure to the impact of reproductive health due to climate change had good knowledge (52.8%), and the students without any prior knowledge did not have good knowledge (44.1%). The primary sources of information include the internet/social media (42.3%), followed by teachers (20.3%).

Conclusion

Nursing students from Bhubaneswar had a moderate to good level of awareness related to the impact of climate change on reproductive health. Prior exposure to knowledge of climate change and its impact on reproductive health, along with formal educational sources, essentially enhanced and impacted the knowledge level. Also, it is essential to formally integrate this topic into the standard nursing curriculum for preparing the upcoming future healthcare professionals in a better manner.

## Introduction

Global climate change has become an important public health challenge with growing impacts on reproductive health. Rising temperatures, air pollution, extreme weather events, and vector-borne diseases can adversely affect fertility, pregnancy, and fetal outcomes, particularly among vulnerable populations. Healthcare professionals play an important role in raising awareness and promoting measures to reduce these health risks [1].

Climate change can negatively affect reproductive health by reducing fertility and increasing the risk of adverse pregnancy and neonatal outcomes. Exposure to air pollution, extreme temperatures, and other climate-based stressors highlights the need for climate-responsive public health and healthcare services [2].

Extreme heat is a major consequence of climate change that increases the risk of adverse pregnancy outcomes, including preterm birth, low birth weight, stillbirth, and impaired fetal growth. Women in low- and middle-income countries are particularly vulnerable to these heat-related risks [3].

Air pollution, aggravated by climate change, increases the risk of infertility, miscarriage, preterm birth, low birth weight, and other adverse pregnancy outcomes. Reducing exposure to air pollutants is essential to protect maternal and reproductive health [4]. Climate-related disasters such as floods, cyclones, droughts, and wildfires can disrupt maternal healthcare services and increase the risk of adverse pregnancy outcomes. These events highlight the need for climate-resilient reproductive healthcare, particularly in low- and middle-income countries [5].

Climate change also affects reproductive health by worsening food insecurity, water scarcity, displacement, and access to healthcare, particularly in low- and middle-income countries. Climate change can adversely affect reproductive health through extreme weather events, disruptions in healthcare access, food and water insecurity, and displacement, particularly among vulnerable populations in low- and middle-income countries [6]. Climate change is increasingly influencing reproductive choices and fertility intentions because of concerns about environmental uncertainty and future living conditions. These concerns are emerging as important factors in reproductive decision-making worldwide [7].

Climate change threatens to widen existing gender-based health disparities in India, with women experiencing disproportionate health consequences because of biological, socioeconomic, and cultural vulnerabilities [8].

Odisha is vulnerable to climate change and associated health risks, and the State Action Plan on Climate Change and Human Health identifies the need to strengthen climate-responsive and sustainable healthcare systems [9]. Awareness of climate change and its effects on health is important for nursing students, who will be future healthcare professionals. Existing evidence indicates that nursing students have varying levels of knowledge regarding climate change and its health impacts, highlighting the need for strengthening climate-health education in nursing curricula [10].

Despite growing evidence on the reproductive health impacts of climate change, limited research has explored nursing students' awareness of these issues, particularly in India. Therefore, this study aimed to assess awareness of climate change and its effects on reproductive health among nursing students in a selected college in Bhubaneswar, Odisha.

## Materials and methods

This study was conducted to assess awareness of climate change and its impact on reproductive health among nursing students in a selected nursing college in Bhubaneswar, Odisha. Data were collected directly from the nursing students using the tool.

Study design: descriptive cross-sectional study

The study included nursing students studying in a selected college in Bhubaneswar, pursuing various courses such as B.Sc. Nursing, GNM Nursing, and M.Sc. Nursing. All the students present, both boys and girls, participated in this study. Informed consent was obtained, and data were collected using the questionnaire tool. Those students who were absent were excluded from the study.

Data collection

Data were collected using a structured and validated tool developed specifically for this study. The tool included a section on sociodemographic characteristics, such as course, semester/year of study, residence, and other relevant factors, and a questionnaire consisting of multiple-choice questions on climate change and its impact on reproductive health to assess the students’ knowledge of the topic.

Inclusion criteria

The nursing students enrolled in the selected nursing college of Bhubaneswar were screened to identify participants meeting the following inclusion criteria for the study group: (1) students pursuing B.Sc. Nursing, GNM Nursing, or M.Sc. Nursing programs; (2) students aged 18 years and above; (3) students available during the period of data collection; and (4) students able to understand and respond to the study questionnaire.

Exclusion criteria

Exclusion criteria were as follows: (1) students who were absent during the period of data collection, (2) students who declined participation in the study, and (3) students with incomplete questionnaire responses. The demographic variables included age, gender, course enrolled, year/semester of course, residence, parental education and occupation, and primary source of information related to climate change and its impact on reproductive health. Then, a set of multiple-choice questions was also administered to assess knowledge of climate change and its impact on reproductive health.

The variables for reproductive health awareness included knowledge regarding the effects of global warming, extreme heat, adverse birth outcomes, water scarcity and its complications, including the chances of occurrence of various infections, and the role of a professional healthcare worker during this time.

Statistical and data analysis

The sample included all eligible students with complete questionnaires available during the study timeframe; thus, no prior sample size calculation was necessary. Data were entered into Microsoft Excel (Redmond, WA: Microsoft Corp.) and analyzed using SPSS Statistics version 25 (Armonk, NY: IBM Corp.) for Windows. Descriptive statistics were employed to summarize the data, with continuous variables represented as means and standard deviations and categorical variables as frequencies and percentages. Associations between selected demographic variables and the level of reproductive health awareness were evaluated using the chi-square or Fisher’s exact test, as appropriate, with a p<0.05 considered statistically significant.

## Results

Aggregate knowledge level

Data were collected from 182 samples, i.e., nursing students of Kalinga Institute of Nursing Sciences from February 17, 2026, to February 22, 2026. The results showed that nearly half (48.4%) of the students had good knowledge, 28% of the students had average knowledge, and 23.6% had poor knowledge. After looking at the various background factors, including age, gender, residence, course enrolled, and parental education, we observe that none of these factors had a statistically significant difference in the knowledge and awareness of students. All the p-values were greater than 0.05, which means that the knowledge levels are the same for all background factors. Table 1 depicts the aggregate knowledge level of students about climate change, and Figure 1 depicts the distribution of knowledge levels among nursing students. Table 2 depicts the aggregate association between sociodemographic variables and knowledge levels.

**Table 1:** Distribution of nursing students according to their level of knowledge regarding climate change and its impact on reproductive health.

Knowledge level	Frequency, n	%
Poor knowledge (0-8)	43	23.6
Average knowledge (9-16)	51	28
Good knowledge (17-24)	88	48.4
Total	182	100

**Figure 1:**
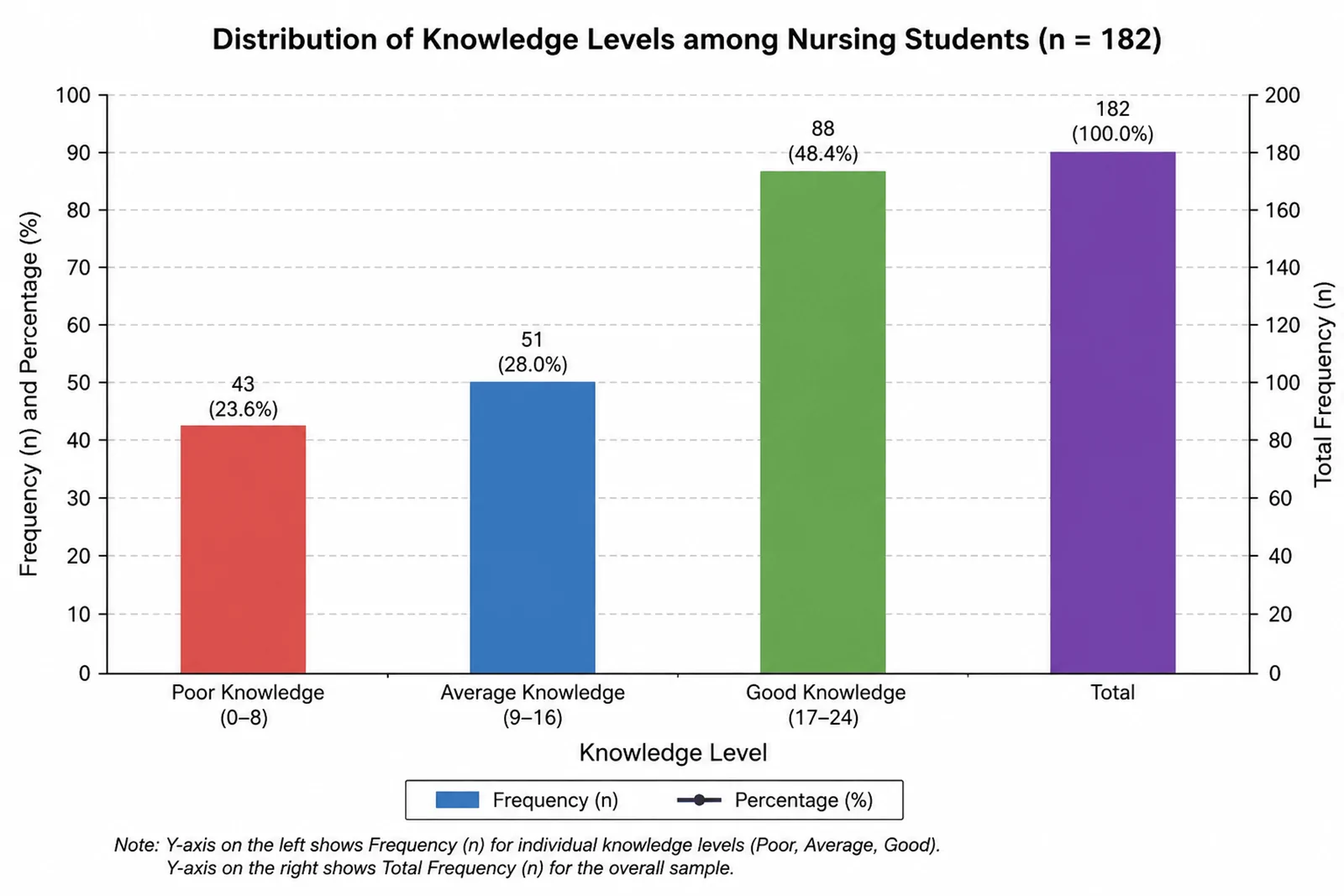
Distribution of nursing students according to their knowledge level.

**Table 2:** Association between sociodemographic variables and knowledge levels regarding climate change among nursing students (n=182). The chi-square test was used for comparison.

Variables	Category	Poor knowledge, n	Average knowledge, n	Good knowledge, n	Total	Chi-square	p-Value
Age	18-19 years	6	6	22	34	11.97	0.062
	20-21 years	13	10	31	54		
	22-23 years	17	23	24	64		
	24 years and above	7	12	11	30		
Total		43	51	88	182	-	-
Gender	Male	27	39	53	119	3.931	0.140
	Female	16	12	35	63		
Total		43	51	88	182	-	-
Course enrolled	M.Sc. Nursing	13	18	25	56	4.629	0.592
	B.Sc. first and second semester	27	31	56	114		
	B.Sc. third and fourth semester	1	1	0	2		
	B.Sc. fifth and sixth semester	2	1	7	10		
Total		43	51	88	182	-	-
Current residence	Urban	20	26	49	95	7.367	0.117
	Rural	18	15	23	56		
	Semi-urban	5	10	16	31		
Total		43	51	88	182	-	-
Family type	Nuclear family	25	33	54	112	3.880	0.422
	Joint family	17	16	32	65		
	Extended family	1	2	2	5		
Total		43	51	88	182	-	-
Head of family occupation	Profession	6	3	6	15	14.360	0.278
	Semi-profession	1	1	3	5		
	Clerical/shop owner/farmer	3	5	2	10		
	Skilled worker	4	10	6	20		
	Semi-skilled worker	12	12	33	57		
	Unskilled worker	2	1	5	8		
Total		28	32	55	115	-	-
Head of family education	Illiterate	2	1	4	7	7.742	0.258
	Primary school	7	8	14	29		
	Middle school	10	9	19	38		
	High school	11	14	20	45		
	Intermediate/diploma	8	10	18	36		
	Graduate/postgraduate	5	9	13	27		
Total		43	51	88	182	-	-
Father's education status	Illiterate	3	2	4	9	10.150	0.254
	Primary school	6	7	13	26		
	Middle school	9	8	16	33		
	High school	12	13	21	46		
	Intermediate/diploma	8	11	18	37		
	Graduate/postgraduate	5	10	16	31		
Total		43	51	88	182	-	-
Mother's education status	Illiterate	4	2	3	9	9.252	0.160
	Primary school	7	6	11	24		
	Middle school	8	9	17	34		
	High school	11	14	24	49		
	Intermediate/diploma	8	10	18	36		
	Graduate/postgraduate	5	10	15	30		
Total		43	51	88	182	-	-
Any prior exposure to climate education	Yes	18	24	47	89	3.334	0.188
	No	25	27	41	93		
Total		43	51	88	182	-	-
Main source of information	Internet/social media	18	20	39	77	5.126	0.274
	Television/radio	9	11	16	36		
	Teachers/college	8	10	19	37		
	Family/friends	5	6	9	20		
	Newspapers/books	3	4	5	12		
Total		43	51	88	182	-	-

Age and gender

The largest proportion of participants (35.2%) were aged between 22 and 23 years. Among different age groups, participants aged 18-19 years had the highest proportion of good knowledge (64.7%). Gender-wise, in this study, the participation of males (65.4%) was higher than that of females (34.6%). A slightly higher percentage of females (55.6%) had better knowledge compared to males (44.5%). Again, gender did not have any significant role in this study.

Course and residence

The maximum number of students (62.6%) were from B.Sc. Nursing first and second semesters. It was observed that students from B.Sc. Nursing fifth and sixth semesters had the highest rate of good knowledge (70%). Almost more than half of the participants were from urban regions (52.2%). It was observed that most participants from urban and semi-urban regions had slightly higher levels of good knowledge (51.6%) than those living in rural regions (41.1%). Here as well, the participants' course and residence did not play a significant role.

Family background and parental education

Almost all participants came from nuclear families (61.5%), and the type of family did not affect the knowledge levels. Among the families where occupation was recorded, 49.6%, i.e., almost half of the heads of the family, were semi-skilled workers. It was observed that neither the head of the family’s education nor occupation significantly affected the knowledge of the participants. It was also observed that the education of the parents did not significantly create any major differences in the results. The percentage of good knowledge ranged from 45% to 50% across all parental education dynamics.

Prior exposure and source of information

Regarding previous learning, all participants were equally distributed in percentage; 48.9% had prior knowledge and exposure, and 51.1% had no prior knowledge and exposure. It was observed that participants with prior exposure scored better (52.8%) with good knowledge compared with participants with no prior exposure (44.1%).

It was also observed that most of the prior information about climate change and its impact on reproductive health was gathered from the internet and social media (42.3%), followed by teachers (20.3%) and TV/radio (19.8%). People acquiring knowledge from teachers (51.4%) or from the internet (50.6%) had the highest rate of good knowledge, while the participants who acquired knowledge from newspapers and books had the lowest score (41.7%).

## Discussion

As we already know, climate change does pose serious threats to worldwide health, with existing reproductive health hazards and broadening gender disparities [10]. It is extremely essential for healthcare professionals to have a basic understanding of climate change and its impact on reproductive health.

The findings of this study revealed that nearly half of the participants (nursing students; 48.4%) had good knowledge of climate change and its impact on reproductive health, while 28% had average knowledge and the remaining 23.6% had poor knowledge. This corresponds with recent systematic reviews conducted globally, revealing that nursing students generally possess a moderate level of awareness and knowledge regarding the impact of climate change on health [8]. Converting this awareness into clinical practice is essential, as healthcare professionals will be dealing directly with climate-related health hazards.

Notably, statistical analyses in the existing studies revealed that sociodemographic factors such as age, gender, residence, parental education, and occupation did not show statistically significant differences in students’ awareness and knowledge levels (p>0.05). Here, in this study, female nursing students did come up with a slightly better (higher) percentage of knowledge (55.6%) as compared to male nursing students (44.5%). Previous studies have shown that women and girls are disproportionately affected by climate change, experiencing higher risks of gender-based violence, reduced access to sexual and reproductive healthcare services, and increased maternal complications during extreme weather events. Climate change can affect the health and well-being of young people, emphasizing the importance of awareness, preparedness, and youth participation in addressing climate-related health risks [11].

In terms of academic advancement, students from the fifth and sixth semesters showed the highest percentage of good knowledge (70%). This shows that as students progress through their advanced nursing curriculum, with increased exposure to clinical practice and academic sessions, they are likely to deepen their understanding of the importance of environmental health problems. Additionally, prior exposure to the issue or subject played a notable role. It was observed that students with prior knowledge of climate change and its impact on reproductive health scored higher (52.8%) than students without prior knowledge (44.1%).

The study found that their primary sources of information were the internet and social media (42.3%), followed by teachers (20.3%) and television/radio (19.8%). Significantly, students with prior knowledge acquired from teachers or the internet did have the highest rate of good knowledge. This emphasizes the crucial role of formal education and digital literacy in promoting climate awareness. To advance from this baseline, the nursing curriculum should formally introduce and include topics on climate change and sustainability to shape and prepare these upcoming healthcare professionals for their professional practice [11].

There are two main limitations to this study. First, the study was conducted at a single nursing college and included 182 students; therefore, the findings may not be generalizable to nursing students at other institutions. Second, the students self-assessed their knowledge, which may not accurately reflect their actual knowledge and may have introduced self-reporting bias.

## Conclusions

This study emphasizes that nursing students in Bhubaneswar had a moderate to good level of knowledge and awareness regarding climate change and its impact on reproductive health. While sociodemographic factors did not significantly affect knowledge levels, prior exposure and sources of information (mainly teachers and the internet/social media) played a crucial role in enhancing their understanding of the topic. As climate change continues to unevenly impact reproductive health and its vulnerabilities, it is essential to formally introduce and integrate climate-related health education into the nursing curriculum. Providing future nurses with this knowledge is necessary for fostering sustainable healthcare systems and improving reproductive healthcare outcomes against the backdrop of global environmental challenges.
